# Unusual inferior dislocation of shoulder: reduction by two-step maneuver: a case report

**DOI:** 10.1186/1749-799X-4-40

**Published:** 2009-11-03

**Authors:** S Saseendar, Dinesh K Agarwal, Dilip K Patro, Jagdish Menon

**Affiliations:** 1 Department of Orthopedics, Jawaharlal Institute of Postgraduate Medical Education and Research (JIPMER), Puducherry, India

## Abstract

Dislocation of the shoulder is the commonest of all large joint dislocations. Inferior dislocation constitutes 0.5% of all shoulder dislocations. It characteristically presents with overhead abduction of the arm, the humerus being parallel to the spine of scapula. We present an unusual case of recurrent luxatio erecta in which the arm transformed later into an adducted position resembling the more common anterior shoulder dislocation. Such a case has not been described before in English literature. Closed reduction by the two-step maneuver was successful with a single attempt. MRI revealed posterior labral tear and a Hill-Sachs variant lesion on the superolateral aspect of humeral head. Immobilisation in a chest-arm bandage followed by physiotherapy yielded excellent results. The case is first of its kind; the unusual mechanism, unique radiological findings and alternate method of treatment are discussed.

## Background

Shoulder dislocations account to 45% of all large joint dislocations[1]. Inferior dislocation of shoulder constitutes 0.5% of all shoulder dislocations[2-4]. Patient characteristically presents with an arm locked in upright position - **Luxatio erecta**[1,5-7]. Its etiology, clinical presentation and roentgenographic findings are distinct. We present an unusual case of recurrent post-traumatic luxatio erecta that transformed later to the adducted position. Such a clinical presentation and recurrence of luxatio erecta have not been described in English literature. The unusual mechanism of injury, unique radiological findings and alternate method of treatment are discussed.

## Case report

**Figure 1:**
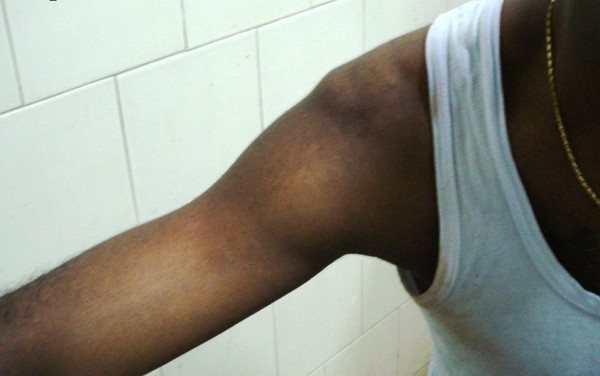
**Pre-reduction clinical picture**. Patient resting arm on table at less than 90deg

**Figure 2:**
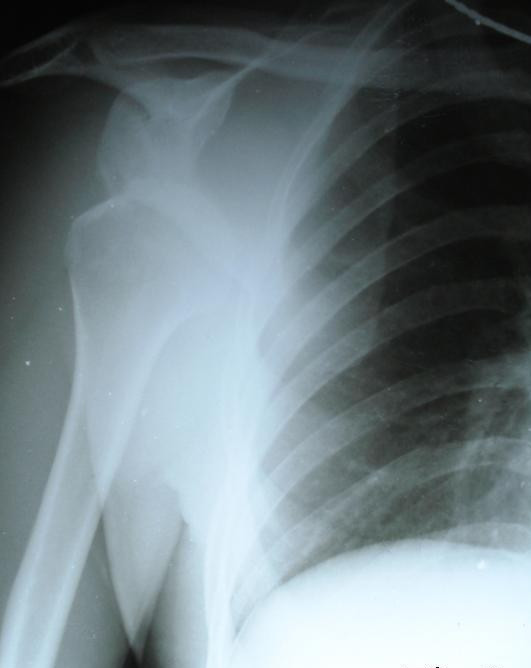
**Pre-reduction radiograph showing subglenoid inferior dislocation with humerus parallel to the chest wall**.

40 year old male athlete presented to the Emergency Department with pain and inability to move right shoulder. His right arm was locked in abduction of 135 degrees. The injury occurred when the patient hyperabducted his arms during a high-jump and presented with the characteristic overhead-abduction of the arm. Examination revealed loss of contour of shoulder, prominence of acromion and presence of subacromion sulcus laterally. Humeral head was palpable in the axilla. There were no neurological deficits. Brachial and radial pulses were palpable. Surprisingly, following analgesia, the patient could rest the arm at less than 90 degrees on a table (Figure 1). During radiography, his arm was parallel to the chest wall (Figure 2).

Detailed history revealed a similar episode of locking of the arm in the abducted position three years before while playing volleyball. However with manipulation by self, the shoulder reduced and pain subsided. General examination revealed features of generalized ligament laxity. Anteroposterior (Figure 2) and modified lateral view radiographs showed inferior dislocation of right shoulder with humerus locked in adduction in the infra-glenoid region.

Under general anesthesia, on repeat examination, the humeral head was still palpable in the axilla and not anteriorly in the shoulder. Closed reduction was carried out based on the two-step maneuver described by Nho et al[8].

### Two-step maneuver for reduction of inferior shoulder dislocation (Figure 3, 4, 5)

**Figure 3:**
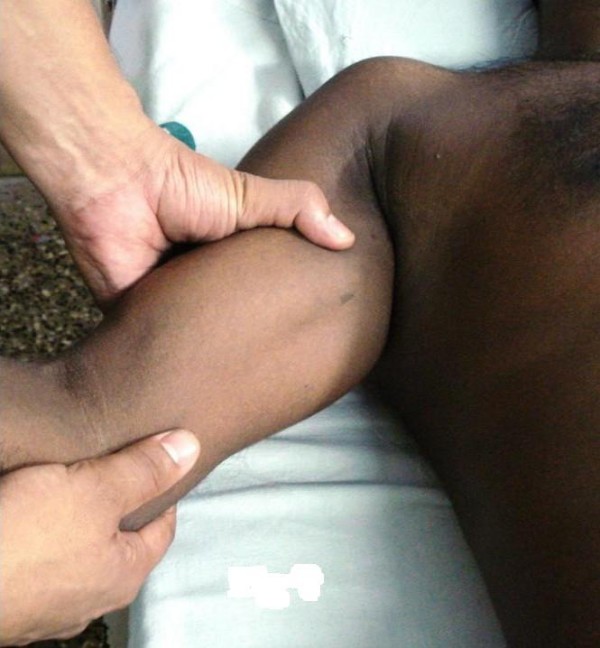
**Demonstration of two-step maneuver: humeral head is levered anteriorly with one hand on the posterolateral aspect of the mid-shaft of the humerus and the other hand positioned over the medial epicondyle**.

**Figure 4:**
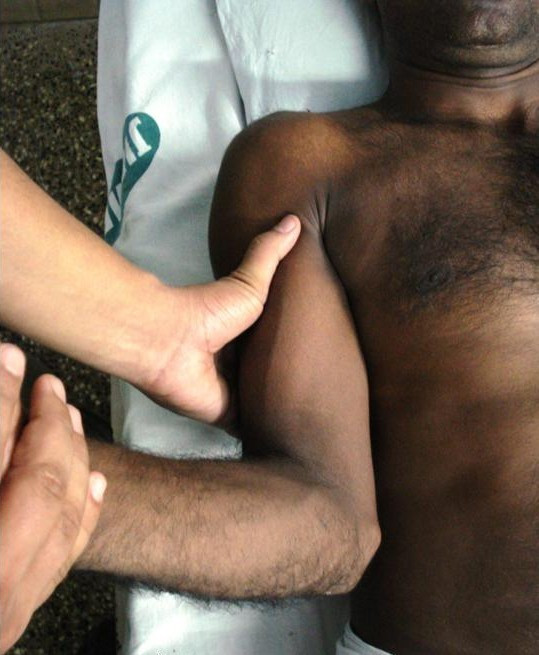
**Demonstration of two-step maneuver: external rotation of arm in adduction reduces humeral head into the glenoid**.

**Figure 5:**
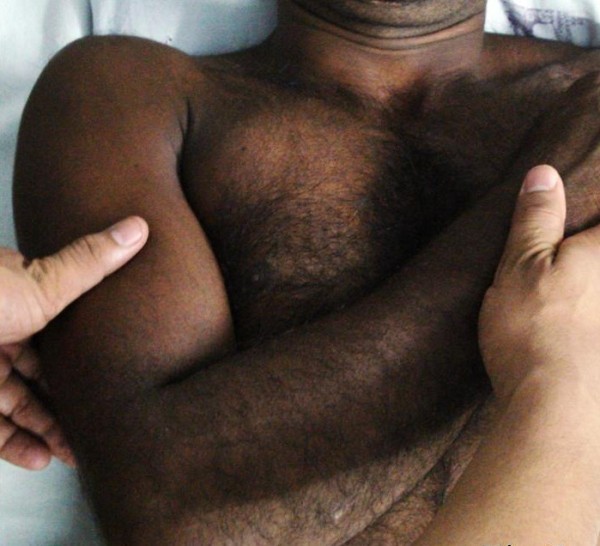
**Demonstration of two-step maneuver: final position of adduction and internal rotation**.

#### Step 1

Patient is positioned supine under sedation or anesthesia. The operator stands on the affected side, by the side of the arm (Figure 3). One arm is placed on the posterolateral aspect of the mid-shaft of the humerus while the other hand is positioned over the medial epicondyle. While the second hand provides mild traction and abduction, the proximal hand gently levers the humeral head from an inferior to an anterior position relative to the glenoid. The first step is complete. Following this step, the humeral head was palpable anteriorly in the shoulder.

#### Step 2

The proximal hand is placed on the lateral aspect of arm to adduct it against the body, while the other hand holds the forearm and externally rotates the arm (Figure 4). The humeral head reduces into the glenoid. The reduction was checked with gentle passive range of motion. The arm was then internally rotated (Figure 5) and shoulder immobilised in a chest-arm bandage.

**Figure 6:**
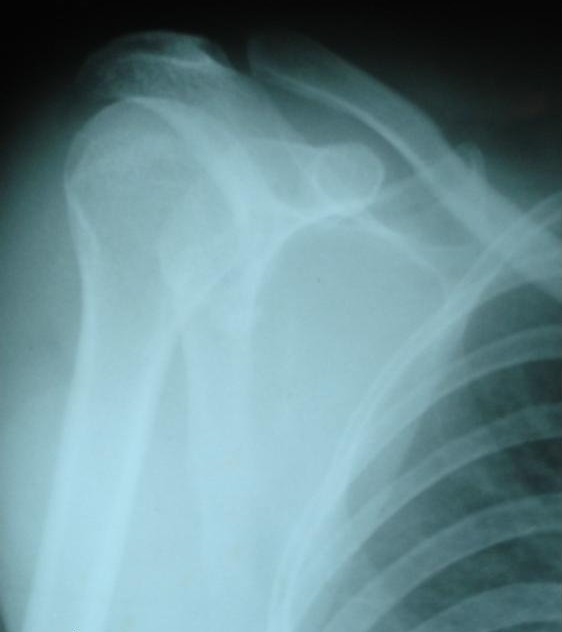
**Post-reduction radiograph showing concentric reduction of shoulder joint**.

**Figure 7:**
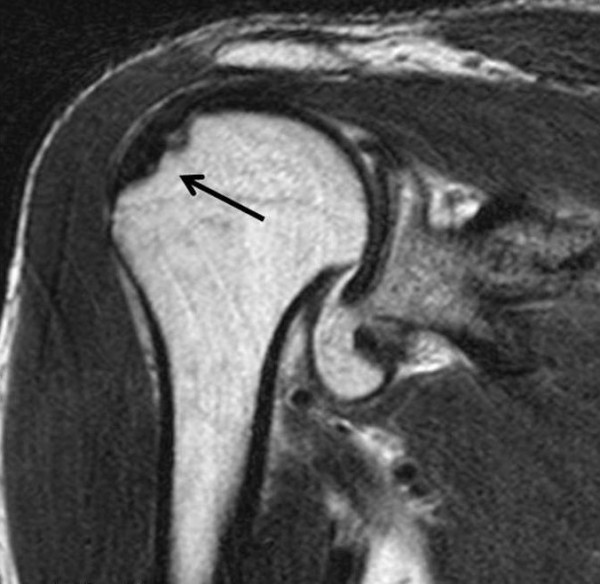
**MRI showing Hill-Sachs variant on superolateral aspect of humeral head(arrow)**.

Post-reduction radiography (Figure 6) demonstrated concentric reduction of joint. MRI to evaluate soft-tissue injury and occult skeletal pathology revealed posterior labral tear and a bony defect on the superolateral aspect (Figure 7). The shoulder was immobilized in a chest-arm bandage for 3 weeks following which he was started on shoulder, elbow and wrist range of motion exercises. At 14 weeks, he regained full range of motion and at last review at 2 years post-trauma, he was still asymptomatic.

## Discussion

True inferior dislocation of the shoulder was first described in non-traumatic disorders such as septic arthritis, stroke and other neuromuscular disorders[9]. Effusion, inferior labral damage and muscular weakness contributed to an inferior subluxation and dislocation in later stages. Traumatic inferior dislocation is a rare injury of the shoulder - the distinct position of the humeral shaft is the most salient feature in making the roentgenographic diagnosis[5]. Based on the location of the humeral head, it can be classified as subglenoid (beneath the inferior rim of glenoid) or subcorocoid (in front of the neck of scapula)[10]. Based on the position of the arm, it can be the luxatio erecta type (humerus parallel to spine of scapula) or true inferior dislocation type (humerus parallel to the chest wall)[9,10].

Two mechanisms of injury have been described for luxatio erecta - direct and indirect[5,11]. In the direct mechanism, there is axillary loading on a fully abducted arm and the humeral head is driven through the weak inferior glenohumeral ligaments and joint capsule, frequently fracturing the greater tuberosity and tearing the rotator cuff. In the indirect mechanism, a violent abduction force on an already abducted limb levers the proximal shaft of humerus over the acromion and the humeral head comes to rest below the glenoid in abduction.

Sonanis et al[9] first reported traumatic inferior dislocation of shoulder without the pathognomonic upright arm posture. A year later Sharma et al[10] reported a similar case of inferior dislocation of shoulder.

### Mechanism of traumatic true inferior dislocation shoulder (Figure 8)

**Figure 8:**
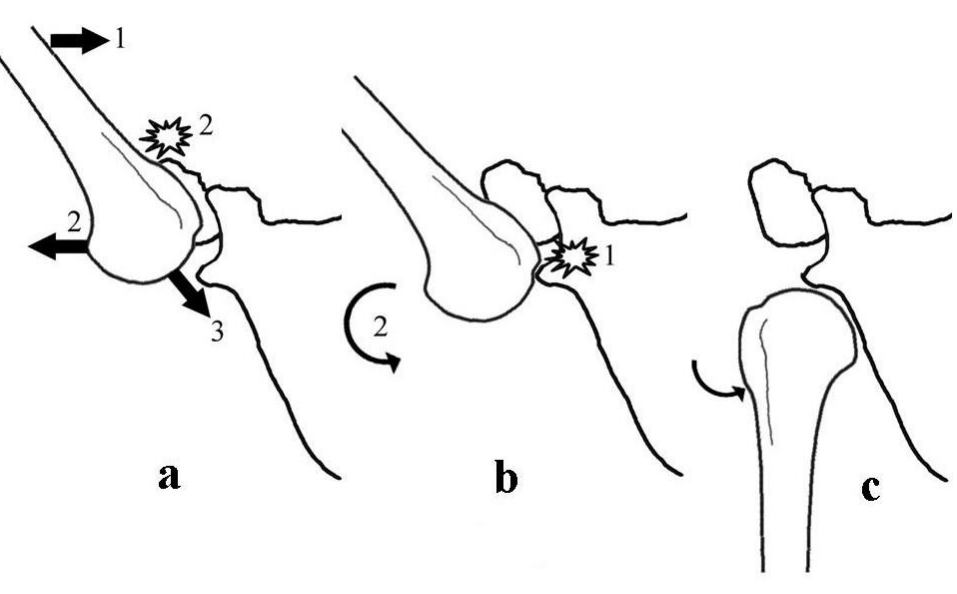
**Figure showing mechanism of true inferior dislocation of shoulder**. a-during hyperabduction proximal humerus is levered over acromion out of the joint; b-inferior glenoid rim impacts on superolateral aspect of humeral head held by muscle spasm; c-humeral head levers over the inferior glenoid rim and comes to rest in the infraglenoid region.

Traumatic true inferior dislocation is also possibly a hyperabduction injury as both patients described so far and the present case all experienced hyperabduction moment of the arm during the injury. Due to the violent abduction force, the proximal humerus is levered over the acromion and the humerus comes to rest in abduction in such a way that the inferior glenoid rim is impacted on the superolateral aspect of the humeral head in the region of the anatomical neck of humerus. Following analgesia and with reduction of muscle spasm, the humeral head gets levered over the inferior glenoid rim and comes to rest in the infraglenoid region in an adducted position. The impingement of head of humerus over inferior glenoid rim results in a bony defect on the superolateral aspect of humeral head. With higher abduction forces, the proximal shaft of humerus comes to rest in apposition to the inferior glenoid rim. The arm in such patients is always locked in abduction and does not change unless manually reduced.

The adducted position of the arm in the present report should not mislead the treating orthopedician to the diagnosis of an anterior dislocation. Attempt to reduce such dislocations by traditional methods for anterior dislocation may result in further trauma. Both the patients described before presented with an adducted arm mimicking anterior dislocation and underwent reduction by the scapulohumeral maneuver developed by Sonanis et al. Nho et al[8] developed the two-step maneuver for reduction of luxatio erecta. He described the method as successful with a single operator, single reduction attempt and minimal force requiring only local analgesia or minimal conscious sedation. In the present report, the two-step maneuver described by Nho et al was used and was successful at first attempt with minimal manipulation.

MRI revealed a variant Hill-Sachs lesion in the superolateral aspect of humeral head and posterior labral tear. Davids and Talbott[5] described a similar lesion by CT scan and stated that the bony defect in inferior dislocation is primarily in the sagittal plane while that in Hill-Sachs lesion is primarily in the frontal plane. Schai and Hintermann[12] and Barnett et al[13] have reported labral injury in inferior dislocation of shoulder. In the present report, immobilization in a chest-arm bandage followed by range of motion exercises resulted in full shoulder function at 14 weeks.

## Conclusion

We present a case of post-traumatic recurrent luxatio erecta humeri, that later transformed unusually to the adducted posture, reduced by the 'two-step maneuver'. The maneuver was successful with single operator, single attempt and minimal manipulation. Closed reduction and immobilisation followed by range of motion exercises resulted in full shoulder function. The mechanism of this subset of inferior shoulder dislocation is different. The bony lesion on the superolateral aspect of humeral head, possibly called a 'cross' Hill-Sachs lesion due to its anatomical relation to the classic Hill-Sachs, appears to be characteristic of inferior glenohumeral dislocation.

## Consent

Written informed consent was obtained from the patient for publication of this case report and any accompanying images. A copy of the written consent is available for review by the Editor-in-Chief of this journal.

## Competing interests

The authors declare that they have no competing interests.

## Authors' contributions

SS - Patient recruitment, treatment, acquisition of data and interpretation of data

SS, DKA, DKP, JM - Treatment and revising the paper critically for important intellectual content. All authors have read and approved the final manuscript.
